# *Akkermansia muciniphila* and Gut Immune System: A Good Friendship That Attenuates Inflammatory Bowel Disease, Obesity, and Diabetes

**DOI:** 10.3389/fimmu.2022.934695

**Published:** 2022-07-07

**Authors:** Vanessa Fernandes Rodrigues, Jefferson Elias-Oliveira, Ítalo Sousa Pereira, Jéssica Assis Pereira, Sara Cândida Barbosa, Melissa Santana Gonsalez Machado, Daniela Carlos

**Affiliations:** Department of Biochemistry and Immunology, Ribeirão Preto Medical School, University of São Paulo, Ribeirão Preto, Brazil

**Keywords:** *Akkermansia muciniphila*, gut dysbiosis, obesity, diabetes, inflammatory bowel diseases

## Abstract

*Akkermansia muciniphila* is a Gram-negative anaerobic mucus-layer-degrading bacterium that colonizes the intestinal mucosa of humans and rodents. Metagenomic data have shown an inverse correlation between the abundance of *A. muciniphila* and diseases such as inflammatory bowel disease (IBD), obesity, and diabetes. Thus, in recent decades, the potential of this bacterium as an immunomodulatory probiotic for autoimmune and chronic inflammatory diseases has been explored in experimental models. Corroborating these human correlation data, it has been reported that *A. muciniphila* slows down the development and progression of diabetes, obesity, and IBD in mice. Consequently, clinical studies with obese and diabetic patients are being performed, and the preliminary results are very promising. Therefore, this mini review highlights the main findings regarding the beneficial roles of *A. muciniphila* and its action mechanisms in autoimmune and chronic inflammatory diseases.

## Introduction

The intestine is mainly colonized by four phyla of bacteria: Firmicutes, Bacteroidetes, Proteobacteria, and Actinobacteria (1). Several factors, such as the use of antibiotics, diet, and pH can interfere with the gut microbiota. It is known that alterations in the gut microbiota (dysbiosis) are capable of inducing abnormal immune responses in the gut-associated lymphatic tissue and that these alterations can compromise the systemic immune response (2). The gut microbiota regulates the host immune response through two main mechanisms: activating the innate immune response *via* the Toll-like receptor (TLR) (3) and/or activating free fatty acid receptors (FFAR) *via* microbial metabolites such as short-chain fatty acids (SCFAs), including acetate, propionate, and butyrate. In addition, these metabolites can induce the differentiation of naive T cells into regulatory T cells (Tregs) or their migration into the intestine (4). Intestinal dysbiosis can lead to excessive activation of TLRs and a low production of SCFAs, contributing to the development of a number of gastrointestinal diseases, obesity, and diabetes (5–7). Because some probiotic bacteria in the gut can suppress chronic inflammatory and autoimmune diseases, the use of probiotics, like Bifidobacteria, Lactobacilli, Lactococci, and Streptococci, as prophylactics and/or therapeutic tools for these diseases has been investigated (8). More recently, *Akkermansia muciniphila* has been shown to be a promising probiotic (9).

*A. muciniphila* is a Gram-negative, anaerobic, oval-shaped bacterium that degrades the mucus layer. The analysis of the 16S rRNA gene sequence showed that this species belongs to the Verrucomicrobia phylum (10). *A. muciniphila* colonizes the intestinal tract early in life and comprises approximately 3% of the total microbiota in healthy adults (11). Upon degrading mucin, *A. muciniphila* produces acetate and propionate, which serve as substrates for other bacteria and the host (10, 12). SCFAs have also been linked to the regulation of body weight gain through their anorexic, anti-inflammatory, and metabolic effects (13, 14). As *A. muciniphila* is lower in the gut of humans and mice with autoimmune and metabolic diseases (15–17), this review highlights the immunomodulatory potential of *A. muciniphila* in these diseases.

## Role of *A. muciniphila* in Protecting Against Inflammatory Bowel Disease

It is known that the impairment of homeostasis and the integrity of the intestinal barrier result in the development of metabolic and gastrointestinal disorders (18, 19). The intestinal mucosal barrier has evolved to maintain a balance between the absorption of essential nutrients and the prevention of pathogen translocation (20). The integrity of the intestinal epithelium is maintained by tight junctions (TJs), adherens junctions (AJs), and desmosome complexes of the epithelium, whose expression can be increased by probiotics or compounds produced by them, such as extracellular vesicles (EVs) or outer membrane microvesicles (OMVs) in the case of Gram-negative bacteria (21–23). The disruption of the integrity of the intestinal mucosa drives the development of inflammatory bowel disease (IBD), such as ulcerative colitis (UC) and Crohn’s disease (CD), which are chronic idiopathic inflammatory diseases characterized by an exaggerated immune response to gut microbiota, resulting in tissue damage (24, 25). It has been reported that antibiotic use during childhood alters the gut microbiota and increases susceptibility to IBD, suggesting an important role of gut microbiota in the maintenance of intestinal homeostasis (26). Several studies have found differences in the composition of the gut microbiota between healthy people and patients with IBD, with a remarkable reduction in *A. muciniphila* in patients with UC (16, 27). Furthermore, it has been reported that *A. muciniphila* or Amuc_1100 (an outer membrane protein of *A. muciniphila*) can attenuate DSS-induced colitis in mice. The modulating effect of *A. muciniphila*-derived Amuc_1100 in colitis was associated with a reduction in infiltrating macrophages, CD8^+^ cytotoxic T lymphocytes, and pro-inflammatory cytokines, such as tumor necrosis factor-α (TNF-α), interleukin (IL)-1α, IL-6, IL-12, macrophage inflammatory protein-1 (MIP-1) α, granulocyte colony-stimulating factor, and chemokine (C-X-C motif) ligand 1 (CXCL1) in the colon. In addition, *A. muciniphila* administration reduced CD16/32^+^ macrophages in the spleen and mesenteric lymph nodes (MLNs) of mice with colitis (28, 29).

A recent study has shown that Amuc_2109, an enzyme secreted by *A. muciniphila*, also attenuated DSS-induced colitis in mice, increasing the expression of TJs and reducing the expression of the NLRP3 inflammasome (30). However, the protective effect of viable *A. muciniphila* against DSS-induced colitis was shown to be dependent of NLRP3 activation (27). Indeed, the role of NLRP3 in the regulation of intestinal homeostasis was previously elucidated, since NLRP3^-/-^ mice are more susceptible to the development of experimentally induced colitis (31).Additionally, it was demonstrated that the administration of *A. muciniphila* induced the proliferation of intestinal stem cells and boosted the differentiation of Paneth and goblet cells in the small intestine and colon of healthy mice or mice with gut damage caused by radiation and methotrexate. In the same study, the beneficial effect of *A. muciniphila* in the intestinal tract was associated with a greater amount of acetic and propionic acids in the cecal content of mice treated with *A. muciniphila* (32), thus demonstrating that this bacterium contributes to the tissue repair of the intestinal mucosa and that the production of SCFAs is involved in this process. Although *A. muciniphila* is a common component of the human and murine gastrointestinal tracts and has a beneficial role for the integrity of the intestinal mucosa, when intestinal dysbiosis occurs, the colonization by *A. muciniphila* can exacerbate the inflammation (11, 33). A previous study reported that treatment with *A. muciniphila* led to the worsening of intestinal inflammation caused by *Salmonella enterica* Typhimurium infection in gnotobiotic mice, which was related to a decrease in goblet cells and an increase in the expression of pro-inflammatory cytokines in the cecum (34). However, recently, Ring et al. (2019) demonstrated that in IL-10-deficient (IL-10^-/-^) mice, which spontaneously develop colitis, colonization by *A. muciniphila* had no effect on intestinal inflammation (35).

Interestingly, several studies have indicated that the outer membrane compounds of *A. muciniphila*, or pasteurized bacteria, have greater therapeutic potential for metabolic, inflammatory, and autoimmune diseases than live *A. muciniphila* (36–38). Notably, Kang et al. (2013) reported a change in the composition of EVs in the feces of mice with DSS-induced UC, such as a decrease in the EVs of *A. muciniphila* and *Bacteroides acidifaciens*. In the same study, OMVs from *A. muciniphila* (AmOMV) suppressed the production of IL-6 in colonic epithelial cells (CT26 cell line) stimulated with OMVs from *Escherichia coli in vitro*, and oral administration of AmOMV, but not viable bacteria, attenuated DSS-induced colitis *in vivo* (36). Additionally, in a murine model of high-fat diet (HFD)–induced intestinal dysbiosis, AmOMVs improved the intestinal mucosal barrier function, increased the expression of TJs and IL-10, and inhibited inflammatory markers in the colon. AmOMVs are also able to reduce intestinal permeability, increase the expression of TJs *via* AMP-activated protein kinase (AMPK), inhibit TLR-4 and interferon-alpha (IFN-α) expression, and increase TLR-2 expression and IL-4 production in Caco-2 cell lines *in vitro* (39, 40). These data indicate that *A. muciniphila* components and their OMVs may be potential therapeutic targets for IBD.

## Role of *A. muciniphila* in Protecting Against Obesity and Metabolic Syndrome

The obesity is related to gut dysbiosis as an imbalance between energy consumption and expenditure favors the prevalence of pathobiont bacteria (41–43). Metagenomic studies have shown that the abundance of *A. muciniphila* is negatively correlated with body weight in humans (15, 44, 45). The analysis of gut microbiota in feces showed that obese and overweight children had reduced *A. muciniphila* concentrations compared with lean children (44). Significant multivariate linear associations using microbiome multivariable association with linear models revealed that the abundance of *A. muciniphila* was negatively correlated with fasting blood glucose levels and the body mass index (BMI), suggesting that these bacteria can act in the control of obesity and diabetes (46). Similarly, the experimental obesity model like mice fed an HFD or high-sucrose diet (HSD) and genetically obese mice (ob/ob) showed a negative correlation with the abundance of the *Akkermansia* genera and adiposity, body weight, liver and adipose tissue inflammation, blood glucose serum, insulin, and triglyceride levels (47–49).

**Figure 1:**
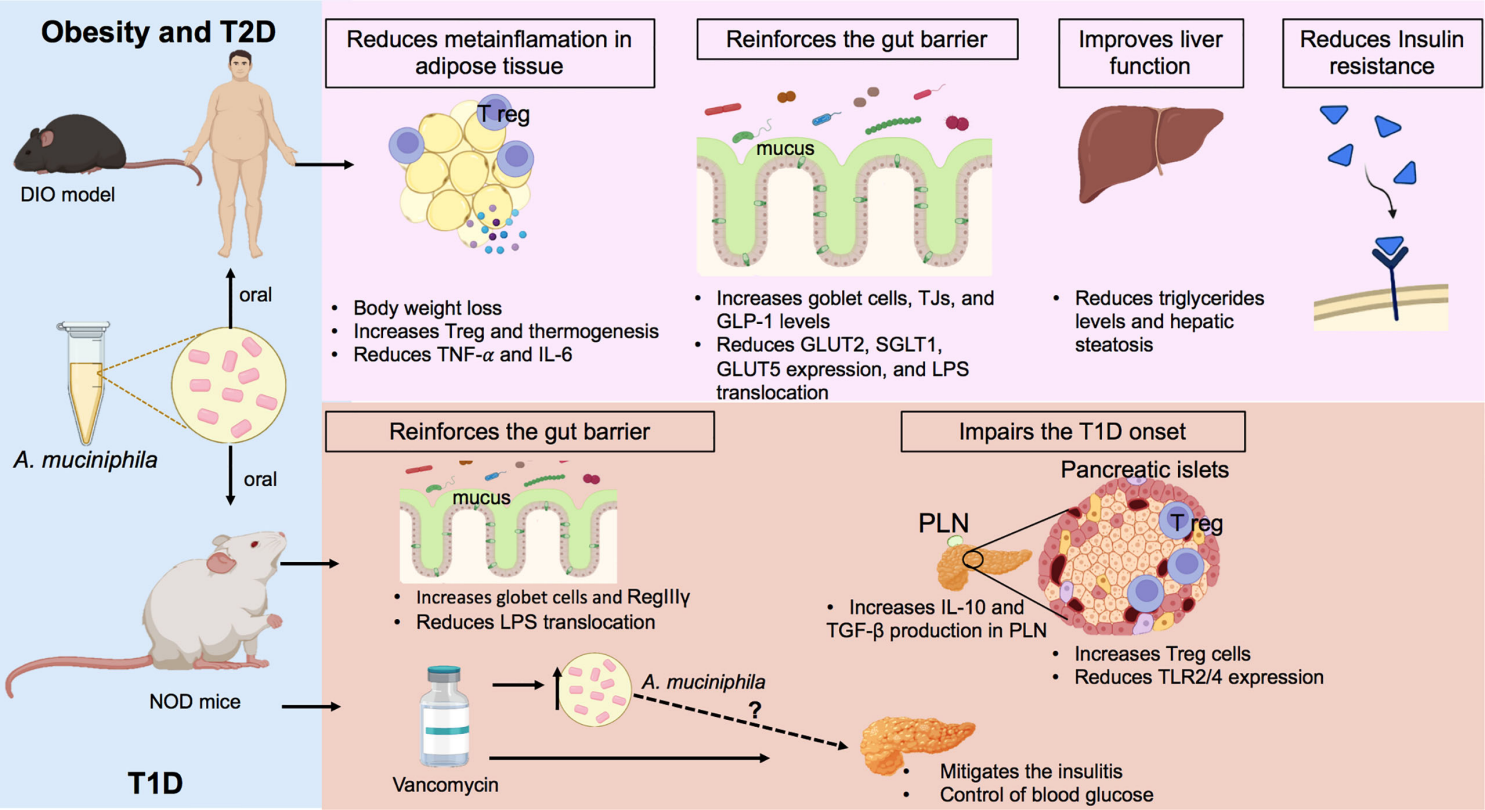
Regulatory effects of *A. muciniphila* on obesity, T2D, and T1D. In the models of diet-induced obesity (DIO), oral treatment with *A. muciniphila* reduces weight gain, controls fat accumulation, increases regulatory T cells (T regs), and decreases the production of pro-inflammatory cytokines in the adipose tissue of mice fed with a high-fat diet (HFD). In the intestine, this probiotic increases goblet cells and mucus production, in addition to inducing a greater production of glucagon-like peptide-1 (GLP-1), which controls glucose absorption. The administration of pasteurized *A. muciniphila* to obese subjects decreased body weight and hip circumference, improved insulin sensitivity, and reduced the markers of liver damage. In NOD mice, the model of T1D (autoimmune), *A. muciniphila* improved mucus production and increased the antimicrobial peptide RegIIIγ, which contributes to improved intestinal barrier function and the lower translocation of LPS into the circulation. In parallel, the probiotic increases the production of anti-inflammatory cytokines in pancreatic lymph nodes (PLNs) and potentiates the recruitment of T regs in the pancreas, culminating in a delay in the development of T1D. Vancomycin-treated NOD mice showed an enrichment of *A. muciniphila* in the gut, which is correlated with a lower degree of insulitis and glycemic control. Figure created with BioRender.com. TNF-α: tumor necrosis factor-α; IL-6: interleukin-6, GLP-1: glucagon-like peptide-1; GLUT2, glucose transporter 2; SGLT1, sodium-glucose cotransporter 1; GLUT5, glucose transporter 5; RegIIIγ: regeneration islet-derived III, IL-10: inyerleukin-10; TGF-β, transforming growth factor beta; TLR2/4, Toll-like receptor 2/4; TJs, tight junctions.

Since there is an inverse correlation between the abundance of *A. muciniphila* and metabolic diseases, several experimental studies have used it as a potential probiotic for therapeutic tools for these diseases (15, 45–47). Similarly, some findings indicate that *A. muciniphila* adheres to the intestinal epithelium and strengthens the integrity of the enterocyte monolayer *in vitro*, suggesting its ability to contribute to the integrity of the intestinal barrier in obese individuals (50). In a murine model of obesity induced by HFD, it was demonstrated that treatment with *A. muciniphila* 10^8^–10^9^ Colony-forming unit (CFU)/mL was able to reduce body weight gain, the accumulation of white adipose tissue (WAT), and energy efficiency and improve liver function impaired by HFD in mice (**Figure 1**) (51, 52). Furthermore, it was reported in the HFD-induced obesity model that the pasteurized form or AmOMV has been successful in reducing or preventing hepatic steatosis and adipogenesis/lipogenesis (38, 53). In addition, the increase in *Akkermansia* ssp. abundance contributed to the antidiabetic effects of metformin in mice fed with HFD, improving glucose tolerance and increasing the population of goblet cells and Treg cells in adipose tissue (54). Kong et al. (2019) reported that intestinal dysbiosis triggered by high-calorie diets in mice can be alleviated by the administration of probiotics that restore proportions and increase the abundance of beneficial bacteria, including *Akkermansia* ssp (55).

The mechanisms by which *A. muciniphila* regulates obesity and glucose levels have not yet been completely elucidated. A previous study showed that *A. muciniphila* was able to increase thermogenesis and the secretion of glucagon-like peptide-1 (GLP-1) and reduce the expression of proteins involved in adipose cell differentiation, and the gene expression of glucose and fructose transporters in the jejunum, suggesting that *A. muciniphila* reduces carbohydrate absorption (**Figure 1**) (52, 56, 57) Similarly, a clinical trial observed that patients who underwent Roux-en-Y gastric bypass surgery showed an increase in the *Akkermansia* population together with an improvement in anthropometric and clinical aspects, such as a reduction in BMI and glycated hemoglobin (HbA1c), as well as an increase in GLP-1 levels (58).

Interestingly, it has been reported that the pasteurization of *A. muciniphila* attenuates metabolic syndrome by reducing body weight, glucose intolerance, insulin resistance, the adipocyte diameter, and the serum levels of leptin and triglycerides (51, 59). In mice fed with HFD, it was also seen that AmOMV showed more suppressive effects, when compared with viable *A. muciniphila*, on lipid metabolism and the expression of inflammatory markers such as TNF-α and IL-6 in adipose tissues (40). Treatment with this probiotic or AmOMV increased the number of goblet cells and TJs in the colon and restored the gut bacterial diversity that has been affected by HFD (40, 51, 59), indicating that *A. muciniphila* can preserve gut homeostasis, which can impact the development of obesity and diabetes. Furthermore, *A. muciniphila*–derived proteins, Amuc_1100 (membrane protein) and P9 (secreted protein), have been reported to reduce obesity-related metabolic syndrome induced by HFD in mice (51, 56). In the case of P9, glucose homeostasis and obesity reduction are related to the interaction of P9 with intercellular adhesion molecule 2 (ICAM-2) and an increase in type 2 macrophages (M2) in an IL-6 dependent pathway (56).

Considering the promising effect of *A. muciniphila* as a therapeutic tool for metabolic syndrome in mice, some clinical studies have been conducted (60–63). In these clinical studies, obese and/or type 2 diabetes (T2D) patients supplemented with *A. muciniphila*, alone or in conjunction with other probiotics, showed improvement in the clinical and metabolic status (**Table 1**). Indeed, a positive effect was also observed in humans, since obese patients who underwent 3 months of treatment with 1 × 10^10^ CFU of pasteurized *A. muciniphila* showed a reduction in weight, fat mass, hip circumference, insulin resistance, plasma cholesterol levels, the markers of liver dysfunction, and systemic inflammation; these patients did not experience any side effects (60). It is important to mention that in addition to the two clinical trials already completed and published (**Table 1**), there are currently two other clinical trials in the recruitment phase, which aim to evaluate the effects of *A. muciniphila* in obesity and T2D (NCT: NCT04797442) and insulin resistance in healthy individuals with dysglycemia (NCT: NCT05114018).

**Table 1:** Recorded and complete clinical trials using *A. muciniphila* administration.

Clinical trial registry number	Type of study	Target disease	Administered *A. muciniphila* preparation	Protocol of administration	Main results	References
NCT02637115	Randomized, double-blind, placebo-controlled pilot study	Overweight/obese insulin-resistant volunteers	10^10^ *A. muciniphila* either live or pasteurized, frozen in glycerol	Daily oral supplementation for 3 months	Improved insulin sensitivity and reduced plasma total cholesterol, fat mass, plasma GTT, AST, LPS, LDH, and creatine kinase	(48)
					*A. muciniphila* counteracted the plasma decrease in 1-PG and 2-PG, endogenous activators of PPARα that may underlie part of the beneficial metabolic effects induced by *A. muciniphila*	(49)
NCT03893422	Randomized, parallel-group, placebo-controlled, double-blind study	Adults with T2D	WBF-011: mixed in capsules, which contained inulin, *Akkermansia muciniphila, Clostridium beijerinckii, C. butyricum, Bifidobacterium infantis*, and *Anaerobutyricum hallii*	Three capsules two times a day within 30 min of morning and evening meals, for 12 weeks	Decrease in total glucose and improvement in glycated hemoglobin	(50)
					Increase in circulating butyrate or ursodeoxycholate, evidencing the need for strategies directed to the microbiome to control T2D	(51)

GTT, γ-glutamyltransferase; AST, aspartate aminotransferase; LPS, lipopolysaccharide, LDH, lactate dehydrogenase; 1-PG, 1-palmitoyl-glycerol; 2-PG, 2-palmitoyl-glycerol; PPARα, peroxisome proliferator–activated receptor alpha.

Furthermore, obese patients with a higher abundance of *A. muciniphila* showed an improvement in metabolic profiles, such as total cholesterol levels and insulin sensitivity after caloric restriction, compared with patients with a low abundance of this bacterium (64). Another clinical trial with overweight and obese diabetic patients showed that inulin and butyrate administration decreased the diastolic blood pressure and the expression of TNF-α levels at the same time exerted bifidogenic effects and increased *A. muciniphila* abundance in these patients (65). Rodriguez et al. (2020) demonstrated that the transplantation of feces from different obese patients to mice with microbiota depleted by antibiotics and fed with HFD differentially responded to inulin supplementation, which was related to the initial gut microbiota composition. In the same study, a positive relationship was observed between an increase in the *Akkermansia* population and weight reduction, increased insulin production, and reduced hepatic and muscle fat in mice supplemented with inulin (66).

The accumulation of visceral fat that occurs in obesity is a risk factor for the development of T2D as its lipolytic activity and greater recruitment of macrophages with a pro-inflammatory phenotype favor insulin resistance (67, 68). In this regard, *A. muciniphila* has been shown to control fat accumulation and adipose tissue metabolism, as well as improve glucose homeostasis by reducing adiposity, fasting glucose, and insulin resistance caused by HFD (15). Studies in diet-induced obese mice have shown that a greater abundance of *A. muciniphila* promoted by the consumption of polyphenols is associated with the prevention of weight gain, reduction of visceral adiposity, prevention of intestinal inflammation, and reduction in circulating liposaccharide (LPS) levels. The results also showed that the increase in the population of *Akkermansia* ssp. improves insulin sensitivity through the reduction of LPS translocation as this bacterium reduces intestinal permeability (69, 70).

## Role of *A. muciniphila* in Resistance Against Diabetes

The ample evidence for the effect of *A. muciniphila* in diabetes is controversial; a case–control two-stage metagenome broad association study with individuals with T2D in China indicated the enrichment of *A. muciniphila* DNA in feces at both evaluation stages (71). However, a variety of metagenomic studies have associated the inverse abundance of *A. muciniphila* in the gut microbiota of obese, prediabetic, and diabetic humans or mice (48, 72–74).Mice fed an HFD for 16 weeks exhibited an increased mRNA expression of inflammation markers in WAT, including *TNF* and *CCL-2*, in addition to developing hyperinsulinemia, hyperglycemia, and higher serum leptin concentrations. All of these metabolic and inflammatory changes were accompanied by a lower abundance of *A. muciniphila* after 3 weeks of diet and precede peripheral insulin resistance and T2D development (48). However, the influence of the relative abundance of *A. muciniphila* does not seem to be only involved in the T2D onset. Refractory T2D is characterized by an individual’s inability to achieve optimal glycemic control, marked by the serum levels of HbA1c less than or equal to 8%. A metagenomic study of stool samples indicated that patients with refractory T2D exhibited a lower relative abundance of *A. muciniphila* than diabetic subjects who achieved optimal glycemic control using metformin or other hypoglycemic agents (75). In an experimental model of T2D induced by streptozotocin (STZ) in rats, viable or pasteurized *A. muciniphila* attenuated T2D, which was associated with improved liver function and reduced plasma pro-inflammatory factors, gluco/lipotoxicity, and oxidative stress (76).

T2D is characterized by changes in glucose metabolism through the resistance of peripheral tissues to insulin, while T1D is an autoimmune disease in which it is possible to observe the progressive destruction of insulin-producing pancreatic β-cells by an inflammatory cell infiltrate and the production of autoreactive antibodies. Despite the differences in the nature of the development of these pathologies, both diseases are accompanied by gut dysbiosis (77–79). In case of T1D, the cytokine IFN-γ has an important role in the diabetes onset (80). Interestingly, IFN-γ^-/-^ mice show better glucose tolerance and increased gut *A. muciniphila* relative abundance compared with wild-type mice. In the same study, IFN-γ^-/-^ mice without *A. muciniphila* showed no improvement in glucose tolerance (81), indicating that the diabetogenic role of IFN-γ may be related to its ability to induce changes in the gut microbiota, especially in the reduction of *A. muciniphila*.

Interestingly, one study indicated that NOD mice that received oral treatment with the antibiotic vancomycin from birth to day 28 of life predominantly have *A. muciniphila* in their gut microbiota, which was accompanied by a lower incidence of T1D. Additionally, mice that received vancomycin treatment from the eighth week of life developed less insulitis associated with reduced destruction of pancreatic β-cells (**Figure 1**) (82). Similarly, diabetes-resistant antibiotic-treated STZ injected mice exhibited an increase in *Akkermansia* abundance (83). These results suggest a protective effect of *A. muciniphila* against T1D development. Furthermore, by observing two colonies of NOD mice with different incidence rates of diabetes, it was possible to notice that the colony of NOD mice with a low incidence of T1D presented a greater abundance of *A. muciniphila* in the gut microbiota. It is worth mentioning that administration of *A muciniphila* to the colony of NOD mice with high-incidence T1D delayed the T1D outcome (**Figure 1**) (84). It was seen that regulation of T1D/T2D by *A. muciniphila* was associated by a local modulation with goblet cell hyperplasia, increased M2 macrophage number and antimicrobial peptide expression in the colon, and the reduction of bacterial translocation. The *Akkemansia*-induced gut homeostasis was accompanied by increased insulin secretion and Treg lymphocyte numbers in the pancreas from diabetic mice models (74, 76, 84). Together, these studies elucidate the role of gut microbiota in the regulation of metabolic and autoimmune diseases, suggesting that probiotic bacteria, especially *A. muciniphila*, can be used as a therapeutic tool against diabetes.

## Conclusion Remarks

Gut dysbiosis has been proposed to be a risk factor for the development of inflammatory and metabolic diseases. Restoring a balanced microbiota and modulating the gut–immune system axis using probiotics has been increasingly studied as a therapeutic strategy for these diseases. It has already been verified that *A. muciniphila* is capable of attenuating metabolic syndrome and damage to the intestinal mucosa by inducing an anti-inflammatory response and controlling intestinal homeostasis. Interestingly, the derived proteins *A. muciniphila*, AmOMV, and pasteurized *A. muciniphila* proved to be more efficient than viable bacteria for alleviating these diseases. Thus, *A. muciniphila* bacterial products or secreted proteins, named as postbiotics, have been proven to be promising targets as new therapeutic tools against chronic inflammatory and metabolic diseases.

## Author Contributions

VR, JE-O, IP, JP, SB, and MM equally contributed to the manuscript writing. DC coordinated and reviewed the manuscript. All authors contributed to the article and approved the submitted version.

## Funding

This study was supported by grants from the São Paulo Research Foundation (FAPESP) (Process numbers: 2018/14815-0, 2020/05514-6).

## Conflict of Interest

The authors declare that the research was conducted in the absence of any commercial or financial relationships that could be construed as a potential conflict of interest.

## Publisher’s Note

All claims expressed in this article are solely those of the authors and do not necessarily represent those of their affiliated organizations, or those of the publisher, the editors and the reviewers. Any product that may be evaluated in this article, or claim that may be made by its manufacturer, is not guaranteed or endorsed by the publisher.
